# Morphological, phytochemical, and pharmacological properties of the genus *Tamarix* in Kazakhstan species: a review

**DOI:** 10.7717/peerj.20059

**Published:** 2025-09-30

**Authors:** Arailym Daulbayeva, Gulnara Kadyrbayeva, Kaldanay Kozhanova, Shazlin Shaharudin, Nurgali Rakhymbayev, Zoya Allambergenova, Rabiga Anarbayeva, Urziya Alimova, Aigerim Kantureyeva, Ainash Baidullaeva, Vladimir An, Bakkonat Kydyrbay

**Affiliations:** 1 School of Pharmacy, S.D. Asfendiyarov Kazakh National Medical University, Almaty, Kazakhstan; 2 School of Health Sciences, Universiti Sains Malaysia, Kota Bharu, Malaysia; 3 Department of Medicine Technology and Pharmacognosy, South Kazakhstan Medical Academy, Shymkent, Kazakhstan

**Keywords:** Antioxidant, Antidiabetic, Anti-inflammatory, Antimicrobial, Taxonomy

## Abstract

The tree-like plants of the genus *Tamarix* belong to the Tamaricaceae family and predominantly grow in subtropical areas, steppes, and saline soils. These plants exhibit various pharmacological properties, including antioxidant, antidiabetic, anti-inflammatory, and antimicrobial characteristics. The current flora of Kazakhstan also includes 13 *Tamarix* species. Despite the diversity of the *Tamarix* taxon, the species discovered in the region have received limited investigation. Therefore, this review analyzed Kazakhstan and international-related scientific studies concerning the morphological, phytochemical, and pharmacological properties of 13 species within the *Tamarix* genus. The plants’ biological features and potential applications were also comprehensively analyzed. Consequently, this review significantly contributed to botanical science and practical pharmacology. Conservation and sustainable employment of the *Tamarix* species for medical purposes could also be developed.

## Introduction

*Tamarix* L. is a genus of deciduous shrubs and small trees belonging to the family *Tamaricaceae*, widely distributed across arid regions of Eurasia and Africa (Bencherif et al., 2020). These plants typically inhabit saline soils, river floodplains, and desert environments, exhibiting high salt tolerance and a remarkable ability to stabilize sandy substrates.

The taxonomy of the genus *Tamarix* is considered highly complex, as its species are morphologically similar, which complicates accurate identification and leads to discrepancies among taxonomists. Historically, up to 200 species and intraspecific taxa of *Tamarix* have been described. However, a revision by B. Baum (1978) reduced this number to 54 species and 15 infraspecific forms (Venturella, Baum & Mandracchia, 2007). More recent data have clarified the taxonomic structure of the genus: according to the online resource *Plants of the World Online* (Royal Botanic Gardens Kew, 2024), the genus currently includes 73 accepted species. Such discrepancies are primarily due to a high number of synonyms and varying interpretations of species boundaries by different authors. For instance, the Russian digital flora reference *Plantarium* considers *Tamarix karelinii* Bunge to be a hybrid (*T. hispida* Willd. × *T. ramosissima* Ledeb.) (Plantarium, 2025), whereas Royal Botanic Gardens Kew (2024) recognizes *T. karelinii* as a distinct species. This highlights the importance of utilizing up-to-date taxonomic databases and incorporating regional floristic sources when clarifying species nomenclature.

In Kazakhstan, *Tamarix* species are predominantly found in desert and semi-desert regions, including the Caspian Lowland, the Aral Sea basin, the Moyynkum and Balkhash deserts, and the river valleys of southern regions. Among them, *T. ramosissima*, *T. hispida*, and *T. laxa* are the most widespread and ecologically significant species (Ile-Balkhash, 2025; The Ferns Database, 2025; Breckle, Wucherer & Dimeyeva, 2012).

Notably, the ecological, medicinal, and economic aspects of the *Tamarix* genus underscore its significance. The *Tamarix* species have also demonstrated resilience in extreme conditions, including high soil salinity and drought. This observation suggests that the plants are valuable subjects for soil stabilization and erosion prevention-related studies. Furthermore, the properties of this species are highly suitable for ecological projects aimed at combating desertification and restoring lands surrounding the Aral Sea (Ashirbekov, 2013; Daryo.uz, 2024).

*Tamarix* species have long attracted scientific interest due to their ecological adaptability and rich phytochemical profile. These plants thrive in arid and saline environments and play an essential role in stabilizing desert ecosystems. Their ability to produce a wide range of secondary metabolites, including polyphenols, flavonoids, and tannins, underlies their demonstrated antioxidant, anti-inflammatory, and antimicrobial activities (Bikbulatova & Korul’kina, 2001; Baktybayeva et al., 2019).

Taxonomically, the genus *Tamarix* is considered complex due to overlapping morphological traits and frequent hybridization among species, which complicates accurate species identification and classification (Sheidai et al., 2018). This complexity is especially relevant in Kazakhstan, where several *Tamarix* species share similar habitats and morphological characteristics.

In Kazakhstan, *Tamarix* plants are also valued for their medicinal applications. Local populations use the bark, leaves, and flowers of *T. ramosissima* and related species to treat digestive disorders, hematological conditions, and infections. Ethnomedical practices across Central Asia, India, and the Middle East describe *Tamarix*-based remedies for ailments such as jaundice, tuberculosis, menstrual irregularities, and various skin conditions (Ibn Sino, 1996; Amirdovlat, 1990; Zakhidov, 1991; Gemedzhieva, Sultanova & Abilov, 2013). These uses highlight the ethnopharmacological significance of the genus in traditional medicine systems.

Studies on the chemical composition and bioactivity of *Tamarix* species in Kazakhstan have confirmed the presence of valuable bioactive compounds, yet few have combined these data with taxonomic assessments (Abilov & Sultanova, 2018). One of the earliest systematic efforts to classify *Tamarix* was made by Joseph Franz Frein in 1903, laying the foundation for future botanical studies on the genus (Villar et al., 2014). Consequently, scientists have advanced their complex structure comprehension of the genus arising from the development of molecular phylogenetics.

Various sources and research methodologies reported differing numbers of plant species in the *Tamarix* genus. Villar, Alonso & Crespo (2022) suggested a taxonomic revision of the genus *Tamarix* into seven species, of which three were native to the Iberian Peninsula and the Balearic Islands. This outcome updated the data of Flora Iberica published in 1993. Sheidai et al. (2018) revealed that the genus *Tamarix* comprised 54 and 90 species, with Asia and the eastern Mediterranean as the primary speciation centers. Akhani et al. (2019) documented a new species (*T. humboldtiana*) in southern Iran. The information confirmed the existence of various species in the region. Several studies also noted approximately 70 species of *Tamarix* that adapted to hot and dry environments (Krylenko, Krylenko & Krylenko, 2020).

Pollen investigations of various *Tamarix* species in Iran revealed diagnostic signs that aided taxonomic identification (Shagholi, Keshavarzi & Sheidai, 2020). This finding presented significant diversity, which complicated species separation. The palynological characteristics of the genus *Tamarix* in Israel were also described in “Pollen Morphology of the Genus *Tamarix* in Israel”, complicating taxonomic clarification of the genus based on palynological attributes (Eshel et al., 2019).

Molecular approaches are vital in distinguishing the taxonomy and phylogeny of the *Tamarix* genus, such as inter-simple sequence repeat (ISSR), random amplified polymorphic (RAPD), deoxyribonucleic acid (DNA), internal transcribed spacer (ITS) sequences, single nucleotide polymorphism (SNP) markers, DNA barcoding, and microsatellites. Hence, incorporating genetic markers and DNA analysis can improve three main processes: (i) accurate species categorization, (ii) hybridization identification, and (iii) practical examination of evolutionary relationships within the genus (Sheidai et al., 2018; Arianmanesh et al., 2015; Lee, Gaskin & Kim, 2019; Terrones et al., 2022). Comprehensive chloroplast genome-related studies can also further expand the understanding of the taxonomic correlations among the species (Wang, Cao & Wei, 2023).

Previous reviews on the genus *Tamarix*, such as those by Bahramsoltani et al. (2020) and Li et al. (2024), have primarily focused on the traditional uses, phytochemical composition, and pharmacological activity of *Tamarix* species at the global level. In contrast, the present review is centered on 13 *Tamarix* species native to Kazakhstan. A comparative analysis of their taxonomic status is presented based on both the international database *Plants of the World Online* (POWO) and regional data relevant to Kazakhstan and Central Asia. The review also integrates ethnopharmacological information on the use of *Tamarix* in Kazakhstan and addresses ecological aspects related to their practical application, including afforestation practices in the Aralkum region.

Therefore, this review aims to summarize the pharmacological potential of these species by examining their morphological characteristics, geographic distribution, phytochemical composition, and known pharmacological properties.

Thus, this study represents an interdisciplinary review that combines taxonomic, ecological, phytochemical, and pharmacological data on *Tamarix* species found in Kazakhstan. It expands the existing body of knowledge and provides a scientific basis for the sustainable use and conservation of these species.

## Survey Methodology

This review conducted a literature search across four scientific databases: (i) Web of Science, (ii) Scopus, (iii) SciFinder, and (iv) PubMed. The keywords employed were “*Tamarix*”, “*Tamarix* morphology”, “*Tamarix* phytochemical compounds”, “*Tamarix* pharmacological properties”, and “*Tamarix* habitats”. The search covered publications from 1951 to 2025, and only peer-reviewed articles and academic sources in English, Russian, and Kazakh were considered.

Only studies focusing on the morphological, phytochemical, and pharmacological properties of *Tamarix* L. plants in the flora of Kazakhstan were included. Additionally, the review incorporated data from authoritative regional sources, such as the *Flora of Kazakhstan*, academic textbooks, and the Plantarium website (https://www.plantarium.ru), which provides high-quality taxonomic and ethnobotanical information.

### Morphological characteristics of plants in the genus *Tamarix*

The genus *Tamarix* encompasses multiple species exhibiting significant morphological variations. These plants in the genus have adapted to moist and frequently saline soils found in arid and semi-arid climates. Generally, *Tamarix* plants manifest as shrubs or trees characterized by leaves that have evolved into scales. The flowers of these plants are collected in dense inflorescences measuring between five cm and 10 cm, blooming during spring, summer, and fall. *Tamarix* seeds also possess plumose tips, facilitating dispersal through wind and water (Akhani et al., 2019; Eshel et al., 2019).

Several characteristic features of *Tamarix* have been demonstrated in its morphological data, such as leaf shape with arrangement, floral organ number with structure, and pollen grain attributes (Sheidai et al., 2018; Eshel et al., 2019). Although a considerable degree of hybridization and phenotypic plasticity complicating identification can occur, this information has enabled species categorization (Arianmanesh et al., 2016; Souddi & Bendi Djelloul, 2020). Numerous species in the genus *Tamarix* also possess synonyms that complicate their systematics, necessitating reevaluation during identification and classification (Villar, Alonso & Crespo, 2022; Gorshkova, 1949; Zieliński, 1994; Assadi, Ramak Maassoumi & Khatamsaz, 1989). Table 1 lists the morphological attributes of the 13 *Tamarix* species (two hybrids) native to Kazakhstan. These species include *T. laxa* Willd., *T. meyeri* Boiss., *T. elongata* Ledeb., *T. passerinoides* Delile., *T. hispida* Willd., *T. leptostachys* Bunge, *T. arceuthoides* Bunge, *T. hohenackeri* Bunge, *T. gracilis* Willd., *T. ramosissima* Ledeb., *T. litwinowii* Gorschk., *T. karelinii* Bunge, hybrid *T. hispida* Willd. × *T. ramosissima* Ledeb., *T. eversmannii* Presl., hybrid *T. leptostachys* Bunge × *T. ramosissima* Ledeb.

**Table 1:** Morphological feature summary of *Tamarix* plants in Kazakhstan.

Species	Morphology	Reference
***1*. *Tamarix litwinowii*** **Gorschek**	**Shrub** height up to 3 metres. **Bark** matt, light brown or grey, smooth. **Leaves:** Leaves of growing shoots: ovoid-elongated, 1–2 mm, acuminate, descending. The leaves of the annual branches are broad, long, acuminate, slightly descending, and protruding. **Flowers:** 4-membered during spring flowering, sometimes 5-membered during autumn flowering, small, less than 5 mm in diameter. It usually blooms twice a year. **Capsule:** narrow, 3 mm long, 1 mm wide, approximately 3–4 times longer than the calyx. **The anthers** are rather blunt.	Pavlov (1963); Kostina, Barabanshchikova & Pavlova (2020); Flora of Turkmenistan (1951)
***2. Tamarix laxa* Willd.**	**Branched** bush, 2–3 metres high, large, branches spreading, glabrous, green and bluish. Phanerophyte. **Bark:** old branches grey; young branches short, reddish-brown, grey-cherry, or grey, brittle. **Leaves** are erect, up to 2 mm, ovate, acuminate at the apex, narrowed to the base, yellowish-green; Racemes are 1–3 cm long, 4–7 mm wide, sparse, almost sessile, and appear early in spring on 1-year-old branches. **Flowers** are pink, in dense racemes (4x1 cm), in turn collected in apical panicles on short peduncles. 4-membered during spring flowering and 5-membered during autumn. **Capsule:** narrow, 3–4 mm long, about 1 mm wide, 5–6 times longer than the calyx. **The anthers** are heart-shaped, slightly acuminate or obtuse, dark purplish.	Pavlov (1963); Flora of Turkmenistan (1951); Afanasyev (2012); Chinese Plant Names (2024); Stankov & Taliev (1949); Baum (1978); Golub Valentin (2008); Shang & Lowry II (2007); Chepinoga et al. (2024); Fungi.su (2024)
***3. Tamarix meyeri* Boiss.**	**Shrub** to 2–4 (8) metres tall, green or bluish-glabrous, with grey, reddish-brown, or brownish-grey bark. **Bark:** old trunks brownish-grey, bark of year-old branches purplish, yellowish, or greenish. **Leaves:** annual branches lanceolate, 3–4 mm long with a semi-stemmed base, brown, obtuse, or slightly acuminate, slightly curved inward, slightly broadened at the base, strongly descending. **Flowers:** 4-membered; bracts narrow-long; lower: blunted with a short truncated blunt end; upper: slightly acuminate. **Capsule:** 5–7 mm long, twice as long as the calyx. **Pollen** grains preprolate, acute.	Souddi & Bendi Djelloul (2020); Pavlov (1963); Flora of Turkmenistan (1951); Stankov & Taliev (1949); Baum (1978); Venturella, Gargano & Mandracchia (2012)
***4. Tamarix elongata* Ledeb.**	**Shrub** height 1–3 metres. shrub with blackish brown bare bark. **The bark** of old trunks is grey; that of the year-old branches is greyish-yellow, pale, or reddish-brown. **Leaves** in the lower part of branches large, broadly linear, lanceolate or oblong-lanceolate, 4 to 9 mm long, 1–3 mm wide, acute, first leaves broad, somewhat heart-shaped, thick or fleshy. **Flowers** are 4-membered; calyx with triangular or ovate, acuminate; membranous lobes along the edge; corolla is large. **Capsule:** 4–6 mm long, 2.5–4 times longer than the calyx. **Anthers:** reddish or orange-yellow, ovoid-conical capsule.	Pavlov (1963); Stankov & Taliev (1949); Baum (1978); Shang & Lowry II (2007)
***5. Tamarix passerinoides* Delile. Synonyms: *Tamarix aucheriana* (Decne. ex Walp.) B.R. Baum, *Tamarix macrocarpa* (Ehrenb.) Bunge**	**Shrub** height 1-2 metres, low spreading tree with bark from reddish-brown to dark purple, young parts are pubescent. **The bark** of old branches is dark brown-grey, with a purple-grayish tint. **Leaves:** strongly auricular, bifurcated, 1.5 to 2.5 mm long, somewhat ovate with spreading tip, 1-2.5 mm long (on old branches 3-3.5 mm long), 1-1.5 mm wide with punctate glands. **Flowers** are five-lobed, pinkish to purple-pink. Inflorescences are simple or loosely folded, usually at the beginning of flowering. The racemes are 2 to 5 cm long and 8 to 10 mm wide. **Capsule:** large, 10–12 mm long, with three leathery valves. **The apical heart-shaped anthers are reddish coloured and acuminate. The pollen** grains were prolate. Tectum: Medium reticulate.	Shagholi, Keshavarzi & Sheidai (2020); Arianmanesh et al. (2016); Gorshkova (1949); Zieliński (1994); Assadi, Ramak Maassoumi & Khatamsaz (1989); Pavlov (1963); Flora of Turkmenistan (1951); Baum (1978); Qaiser (1983); De Martis, Loi & Polo (1984); Elkordy & Faried (2017); Qaiser & Perveen (2004)
***6. Tamarix gracilis* Willd.** **Synonyms: *Tamarix affinis* Bunge, *Tamarix polystachya* Ledeb.**	**Shrub** height of 1.5–4 metres. Shrub tree with brown to blackish-brown bark, young parts completely glabrous or with papillae. **The bark** is greyish-green or brownish-chestnut, with grey-pale cork spots on the leaf axils and along the stem. **The leaves** are oval-lanceolate and acute. Flowering racemes are thin, 2.5 to 5 cm long, sparse, and on bare stalks. sessile, with a narrow base, much longer than wide, 1–3 mm long. **Capsule:** 4–6(8) mm long, nearly four times longer than the calyx. **Spring flowers** are 4-membered; summer flowers are 4–5-membered, up to 5 mm in diameter; calyx lobes are ovoid, green, with a serrated translucent edge, obtuse, rarely acuminate, and without a keel. **Pollen:** unit of distribution: monads. ** Size**: medium. Sculpture: mesh. Number of apertures: 3. Aperture position: equatorial. Aperture type: slit.	Shagholi, Keshavarzi & Sheidai (2020); Gorshkova (1949); Zieliński (1994); Assadi, Ramak Maassoumi & Khatamsaz (1989); Pavlov (1963); Stankov & Taliev (1949); Baum (1978); Shang & Lowry II (2007)
***7. Tamarix hispida* Willd.**	**Shrub** height of 1.5–5 metres. A small tree with reddish brown bark, usually very hairy to almost bare, at least with papillae-like brush top bark of old branches and trunks reddish-grey; annual branches and growing shoots with reddish or ochre-grey bark densely covered with short trichomes. **Leaves are** sessile, cordate-cylindrical at the base, 1.5–2.25 mm long. Leaves of annual branches narrowly lanceolate, acuminate, leaves of green shoots ovate-lanceolate, 0.52 mm long, acute, with a point curved inward, semiclasping, pubescent. **Flowers:** 5-membered: calyx 1–1.3 mm long, with broad, obtuse, or pointed lobes, without keel, with a broad, transparent, serrated edge. **Capsule:** 4–5 mm long, 4–5 times longer than the calyx. **The anthers** are rather blunt.	Arianmanesh et al. (2016); Pavlov (1963); Flora of Turkmenistan (1951); Stankov & Taliev (1949); Baum (1978); Shang & Lowry II (2007)
***8. Tamarix leptostachys* Bunge**	**Shrub** height of 1–5 metres shrub or very small tree, tall, with brown bark; young parts glabrous, sometimes with sparse fibrous-papillate leaves. The bark of the old branches is reddish-grey; annuals are grey-purple or reddish. **Leaves are** sessile with a narrow base, 2–3 mm long. The younger leaves are somewhat ribbed, 2–4 mm long, 0.5–2 mm wide, and acute. **The flowers** are five-petalled and pink. The petals are almost free, ovate or tubular-ovate, acute to obtuse, finely serrated along the edge, 0.5–0.75 mm long, 0.25–0.4 mm wide. **Capsule:** elongate-pyramidal, about 1.8 mm long, twice as long as the calyx. **Pollen** grains to prolate. Tectum: Finely reticulate. ** Pollen** grainswith columellae	Pavlov (1963); Baum (1978); Shang & Lowry II (2007); Qaiser (1983); Qaiser & Perveen (2004); Plantarium plant Determinant Online (2024)
***9. Tamarix arceuthoides* Bunge**	**Shrub** height of 1–4 metres. Very small tree, 2–3 (-4) m tall, with reddish-brownish-pinkish bark, densely branched and glabrous. **The bark** of the trunks and old branches is grey, the bark of young woody ones is reddish-brown, dark purplish, or brownish-brown, and and the annual shoots are emerald green, rarely syzy-green, glabrous, thin, straight and ascending. **The leaves** are sessile with a narrow base, usually with rough papillate edges, 1–2.5 mm long. Racemes are 1.5 to 5 cm long and 3 to 4 mm wide. **Flowers** 5-membered; calyx 0.5 to 0.7 mm long; bracts as long as the peduncle or slightly longer; entirely herbaceous; from linear to triangular to narrowly ovate; acuminate at the apex. **Capsule:** 4–6 times longer than the calyx lobes appressed to the capsule. **The pollen** grains were prolate. Tectum: Finely reticulate. ** The anthers are cordate, yellowish**.	Akhani et al. (2019); Shagholi, Keshavarzi & Sheidai (2020); Arianmanesh et al. (2016); Pavlov (1963); Baum (1978); Shang & Lowry II (2007); Qaiser (1983); Qaiser & Perveen (2004); Plantarium plant Determinant Online (2024); Zhang et al. (2018); Ovchinnikov (1981)
***10. Tamarix hohenackeri* Bunge. Synonyms: *Tamarix rosea* Bunge** ***Tamarix smyrnensis* Auct.**	**Shrub** height 1-3 m. Bushes with cranberry bark and linear, blunt, slightly curved. **The bark** of the old branches is greyish-brown; bark of the year old shoots is dark purple. **Leaves** linear or ovate-lanceolate, 1 to 5 mm long, long acuminate, acuminate, pointed, with the tip curved inward, semi-stemmed, without keel; racemes lateral, 1 to 8 cm long. **Flowers** are 4-membered, peduncles are long, calyxes, and flower brushes are yellowish elongated, 4 to 10 cm long and 5 to 6 mm wide, dense, and slightly widened at the base. **Capsule:** 4–5 mm long, three times shorter than the ovary. **The anthers are blunt**.	Gorshkova (1949); Zieliński (1994); Assadi, Ramak Maassoumi & Khatamsaz (1989); Pavlov (1963); Kostina, Barabanshchikova & Pavlova (2020); Flora of Turkmenistan (1951); Stankov & Taliev (1949); Shang & Lowry II (2007).
***11. Tamarix ramosissima* Ledeb. Synonyms: *Tamarix altaica* Nied.** ***Tamarix odessana* Steven ex Bunge** ***Tamarix pallasii* Desv.** ***Tamarix pentandra* Pall.**	**Shrub** height 1–5 m. Shrub or low-growing tree. Phanerophyte, hyperhalophyte **The cork** of old trunks and branches is dark grey; the bark of year old shoots is reddish or orange-reddish. **Leaves** with small sessile green or glaucous scale-shaped leaves that secrete salt on their surface. **The flowers** are small, with a calyx and a pink corolla of four to five petals. Flowers are collected in large clusters. The bracts are equal to the pedicel. **Capsule:** trigonous-pyramidal, 3–5 mm long, 3–4 times longer than the calyx. **Pollen** grains to subprolate, microreticulate. **The anthers are rather blunt**.	Akhani et al. (2019); Shagholi, Keshavarzi & Sheidai (2020); Arianmanesh et al. (2016); Gorshkova (1949); Zieliński (1994); Assadi, Ramak Maassoumi & Khatamsaz (1989); Pavlov (1963); Kostina, Barabanshchikova & Pavlova (2020); Flora of Turkmenistan (1951); Afanasyev (2012); Shang & Lowry II (2007); Qaiser (1983); Qaiser & Perveen (2004); Zhang et al. (2018); Heywood et al. (2007); Crins (1989); Gaskin & Schaal (2003); Gaskin et al. (2004); Britannica (2019).
***12. Tamarix karelinii* Bunge** **Hybrid *Tamarix hispida* Willd.× *Tamarix ramosissima* Ledeb.** **Synonyms: *Tamarix hispida* var. *karelinii* (Bunge) B. R. Baum**	**Shrub** height 2–4 m. **The bark** of the trunks is grey; bark of annual branches are grey-purple; branches glabrous, rarely slightly rough. **The leaves** are simple, sessile, ovoid, covered with salt glands, 1–2 mm long, 0.5–1 mm wide, acute. **Flowers** are pink to dark pink, five-lobed, open or semi-open, almost sessile; the calyx is approximately 1 mm long, with rounded, obtuse, membranous lobes along the edge. **Capsule:** 5–6 mm long, 5–6 times longer than the calyx. **Pollen** grains to subprolate. Tectum: Medium -coarse reticulate. ** Anthers** with short pointed tips.	Gorshkova (1949); Zieliński (1994); Assadi, Ramak Maassoumi & Khatamsaz (1989); Pavlov (1963); Kostina, Barabanshchikova & Pavlova (2020); Golub Valentin (2008); Qaiser (1983); Qaiser & Perveen (2004); Plantarium plant Determinant Online (2024)
***13. Tamarix eversmannii* Presl.** **Hybrid *Tamarix leptostachys* Bunge** × ***Tamarix ramosissima* Ledeb.**	**Shrub** height 2–3 m. **The bark** of old trunks is reddish-grey; the bark of one-year-old shoots is reddish-brown. **Leaves** areovate-lanceolate or ovate-oblong, 1.5–4 mm long, rounded at the base, descending, acute at the apex, usually bent inward; racemes apical, 1–3(4) cm long, 2–3 mm wide, sparse, in a sparse, spreading panicle. **Flowers** are 5-membered; calyx 1 mm long with triangular-ovate, pointed membranous lobes at the edge. **Pollen** grains are pointed; styles 3(4), twice as short as the ovary.	Pavlov (1963)

### Geographical distribution

The plants within the *Tamarix* genus are correlated through a complex taxonomic network. These plants are among the most studied and widespread genera worldwide. *Tamarix* species can also adapt to different ecological conditions, and these plants can thrive in the arid and semi-arid regions of Eurasia. One considerable example involves the primary centers of speciation for the genus *Tamarix* in Central Asia (Kazakhstan, Afghanistan, Iran, and Turkmenistan) (Sheidai et al., 2018). These species have also adapted to saline soils and wet conditions along rivers and oases across Middle Eastern regions (Iran, Iraq, and Saudi Arabia). Similarly, eastern European *Tamarix* plants grow along rivers and in salt marshes within Ukraine and southern Russia. These species of the genus *Tamarix* are also discovered along the coasts, rivers, and lakes of North Africa, indicating high adaptability to arid environments and saline soils (Terzoli et al., 2013; Zhang et al., 2003).

*Tamarix* species (such as *T. ramosissima* and *T. chinensis*) were initially introduced to North America in the 19th century. Nevertheless, these plants exhibit invasive characteristics in the southwestern states of the United States (Arizona, California, Nevada, New Mexico, and Texas). The species thrives along rivers and reservoirs, modifying ecosystems and competing with native flora (Abby Brownell, 2013; iNaturalist contributors, 2024e). In Kazakhstan, Tamarix species play a significant role in desert and semi-desert ecosystems. They are primarily distributed in the Caspian Lowland, the Aral Sea region, the Moyynkum and Balkhash deserts, and the river valleys of southern Kazakhstan, where they form dense thickets in floodplain forests (tugai) and on saline soils. The most common and widely distributed species are *T. ramosissima* Ledeb., *T. hispida* Willd., and *T. laxa* Willd. (Ile-Balkhash, 2025). These three taxa constitute the dominant component of tamarisk thickets across much of their range, from the western deserts to the southeastern regions of the country.

In contrast, other species have more localized distributions. For example, *T. arceuthoides* Bunge is known from southwestern Kazakhstan (Caspian region) (The Ferns Database, 2025), while *T. aralensis* Bunge is primarily confined to the Aral Sea basin. Such narrowly distributed species, often restricted to drying lakebeds or specific desert zones, may be considered potentially vulnerable under changing environmental conditions.

Regional shifts in *Tamarix* species dominance have also been observed. In the desiccated zones of the Aral Sea, *T. laxa* and *T. elongata* dominate older desiccation sites (since the 1960s), *T. hispida* prevails in areas desiccated in the 1980s, and *T. ramosissima* is more common in river deltas (Breckle, Wucherer & Dimeyeva, 2012). This species in particular demonstrates a remarkable capacity to adapt to arid conditions and saline soils (Sheidai et al., 2018).

**Figure 1:**
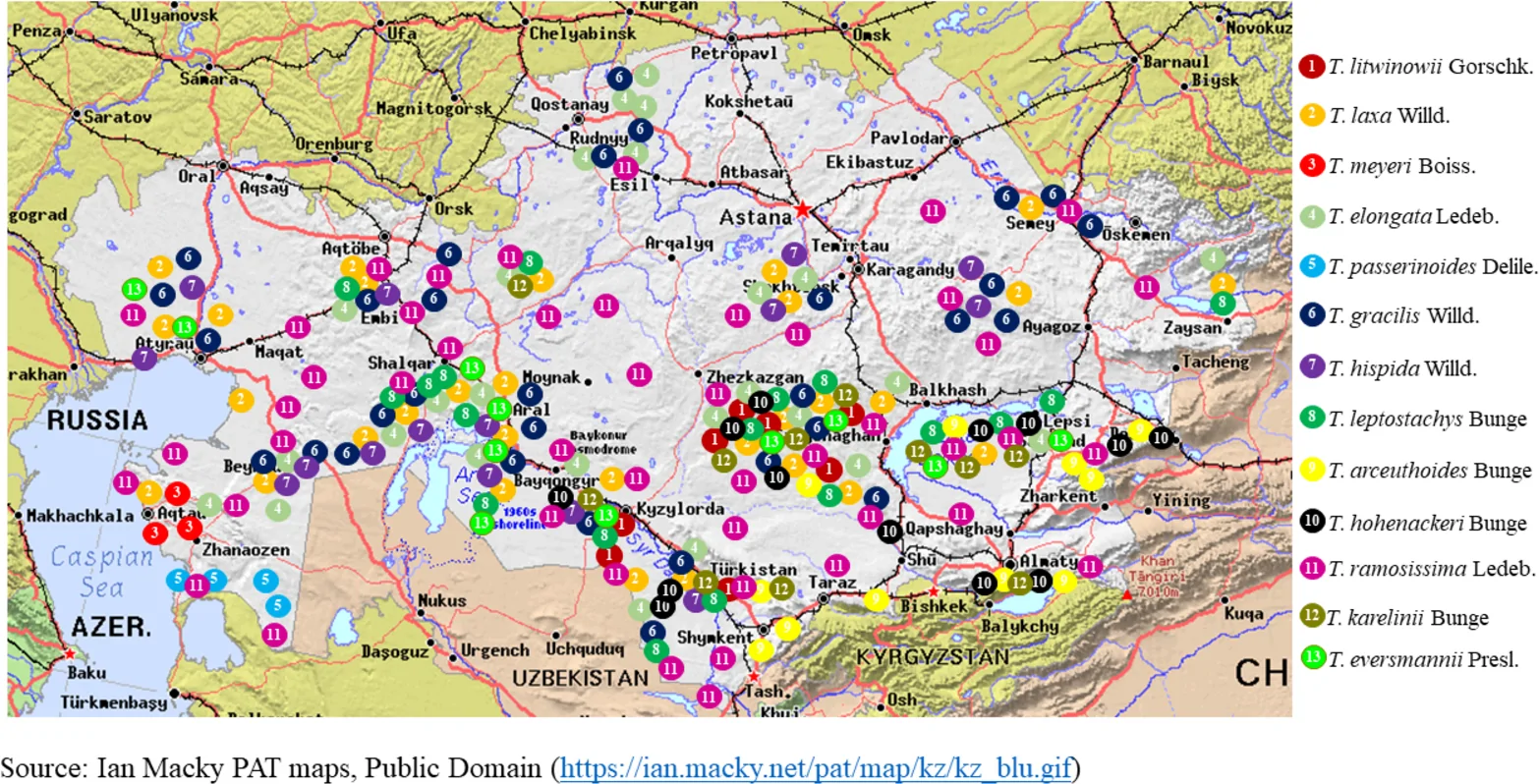
Geographical distribution of *Tamarix* species in Kazakhstan.

Figure 1 illustrates the habitat map of the *Tamarix* species in Kazakhstan. Table 2 presents the global and regional distribution of *Tamarix* species in Kazakhstan, structured according to the floristic zoning system proposed by Rachkovskaya, Khramtsov & Volkova (2003).

**Table 2:** Geographical distribution of *Tamarix* L.

**No**	**Species**	**Geographical** ** distribution** ** around the world**	**Geographical** ** distribution** ** in** ** Kazakhstan**
**1**	***Tamarix litwinowii***	Central Asia, Eastern Iran, Northern Afghanistan, Turkmenistan (Pavlov, 1963)	Kyzylorda, Betpakdala, Muyun-Kum, Kyzylkum, Turkestan (Ile-Balkhash, 2025)
**2**	***Tamarix laxa*** ** Willd.**	Turkmenistan, Caucasus, Central Asia, Iran, Afghanistan, Western China, Mongolia (Pavlov, 1963)	Semipalatinsk, near the Caspian, Aktobe, Embi, Turgen, Western and Eastern Melkosop. Zaisan, Northern Ust-Urt, Mangyshlak, Priaral, Kyzylorda, Betpakdala, Muyun-Kum, Kyzylkum, Turkestan, Balkhash, Alakol (Pavlov, 1963)
**3**	***Tamarix meyeri* Boiss**.	Turkmenistan, Caucasus, Central Asia, Iran, Minor Asia, Syria, Egypt, Tajikistan, Afghanistan (Pavlov, 1963)	Mangyshlak (Pavlov, 1963)
**4**	***Tamarix elongata*** ** Ledeb**	Russia, Kazakhstan, Turkmenistan, Uzbekistan, Central Asia, Western China, southern Mongolia (Pavlov, 1963)	Tobyl, Yeshim, Embi, Turg., Western Melkosop. Zaisan, Northern Ust-Urt, Mangyshlak, Aral, Kyzylorda, Betpakdala, Muyun-Kum, Kyzylkum, Turkestan, Balkhash, Alakol (Pavlov, 1963)
**5**	***Tamarix passerinoides*** ** Delile.**	Turkmenistan, Iran, Asia Minor, Arabia, Egypt, Algerian Sahara, Libya, Sinai (Gorshkova, 1949; Zieliński, 1994; Assadi, Ramak Maassoumi & Khatamsaz, 1989)	South Usturt (Pavlov, 1963)
**6**	***Tamarix gracilis*** ** Willd.**	Western Siberia, Western China, Mongolia, Kazakhstan, Tajikistan, Turkmenistan, Turkey (Gorshkova, 1949; Zieliński, 1994; Assadi, Ramak Maassoumi & Khatamsaz, 1989; Pavlov, 1963)	Tobyl Eshim, Irtysh, Semey, near the Caspian, Mugodzhar, Emb, Turk, Western and Eastern Melkosop, Northern Ustyurt, Aral, Kyzylorda, Betpakdala, Muyun-Kum, Kyzylkum, Turkestan (Yany Kurgan station) (Pavlov, 1963)
**7**	***Tamarix hispida*** ** Willd.**	Central Asia, Afghanistan, China, Mongolia, Kazakhstan, Russia, Uzbekistan, Turkmenistan, Afghanistan (Pavlov, 1963)	near the Caspian, Embi, Turg., Western and Eastern Melkosop, Northern Ustyurt, Aral, Kyzylorda, Turkestan (Pavlov, 1963)
**8**	***Tamarix leptostachys*** ** Bunge**	Central Asia (northern part), Western China, Mongolia (Pavlov, 1963)	Embi, Turg., Zaysan, Aral, Kyzylorda, Betpakdala, Muyun-Kum, Kyzylkum, Turkestan, Balkhash, Alakol (Pavlov, 1963)
**9**	***Tamarix arceuthoides*** ** Bunge**	Central Asia (Tien Shan, Pamir-Alai, Kopetdag), Iran, Iraq, Afghanistan, Western China, Mongolia, Kazakhstan, Turkmenistan, Uzbekistan, Kyrgyzstan, Pakistan, Kashmir (Pavlov, 1963)	Muyun-Kum, Balkhash, Dzungar Alatau, Zail. Kung. Alatau, Kirg Alatau, Karat, west of the TSh (Pavlov, 1963)
**10**	***Tamarix hohenackeri*** ** Bunge**	Caucasus, Central Asia, Northern Iran, Mongolia, Armenia, Azerbaijan, China (Gansu, Jiangxi, Nei-Mongol, Zizhiqiu, Ningxia-Hui, Qinghai and Xinjiang), Crimea, Kazakhstan, Republic of Georgia, Russia, Turkey, Greece (Bikbulatova & Korul’kina, 2001; Xing et al., 2014, Shishkin, 1949; Tropicos, 2024; EUNIS, 2018)	Kyzylorda, Betpakdala, Muyun-Kum, Kyzylkum, Turkestan, Balkhash, Alakol, Dzungar Alatau, Zail. Kung. Alatau, Chu-Il mountains (Pavlov, 1963)
**11**	***Tamarix ramosissima*** ** Ledeb.**	Caucasus, Central Asia, Iran, Iraq, Asia Minor, Afghanistan, China, Mongolia, Ukraine, Kazakhstan, Tajikistan, Uzbekistan, Kyrgyzstan, Azerbaijan, Turkmenistan, Tibet, China, Korea, Armenia, Georgia, Pakistan, Qatar, Turkey, Ukraine, Uzbekistan (Gorshkova, 1949; Zieliński, 1994; Assadi, Ramak Maassoumi & Khatamsaz, 1989; Qaiser, 1983; Shishkin, 1949; Gaskin & Schaal, 2002; CABI International, 2024)	Tobyl Eshim, Semipalatinsk, Prikaspi, Aktobe, Zaysan, Northern Ust-Urt, Mugodzh, Embi, Turg, Western and Eastern Melkosop, Betpakdala, Muyun-Kum, Balkhash-Alakol, Southern Ust-Urt, Kyzylkum, Turkestan, Dzungar Alatau, Chu-Il Mountains, Karat (Pavlov, 1963)
**12**	***Tamarix karelinii* Bunge** (Hybrid *Tamarix hispida* Willd. × *Tamarix ramosissima* Ledeb.)	Central Asia, Iran, Mongolia (Gorshkova, 1949; Assadi, Ramak Maassoumi & Khatamsaz, 1989)	Turg., Kyzylorda, Betpakdala, Balkhash-Alakol, Turkestan, Zail. Kung. Alatau, Karatau (Pavlov, 1963)
**13**	***Tamarix eversmannii* Presl**. (Hybrid *Tamarix leptostachys* Bunge × *Tamarix ramosissima* Ledeb.)	Southeast Europe (Pavlov, 1963)	near the Caspian, Aral, Kyzylorda, Muyun-Kum, Balkhash-Alakol (Pavlov, 1963)

### Phytochemical compounds

The compositions of the varying *Tamarix* species have been significantly investigated. One prominent example includes biologically active compounds in specific species, such as *T. hohenackeri* Bunge and *T. ramosissima* Ledeb. Consequently, all assessed species have shown the presence of flavonoids, phenolic acids, and triterpenoids. Certain species have also revealed other classes of phytochemical compounds, including coumarins, anthraquinones, tannins, tetraterpenes, and steroids (Bikbulatova & Korul’kina, 2001; Xing et al., 2014; Bahramsoltani et al., 2020; Umbetova et al., 2004a; Sultanova et al., 2002; Sultanova et al., 2004b; Utkin, 1966; Ren et al., 2019; Hong et al., 2018; Sultanova et al., 2001; Umbetova et al., 2005; Zhang & Tu, 1994; Sultanova et al., 2004a; Orabi et al., 2013; Xiao et al., 2013; Umbetova et al., 2004b; Sultanova et al., 2013; Yao et al., 2017). This data suggests that the genus *Tamarix* is rich in phytochemical compounds and exhibits various biological activities (antioxidant, anti-inflammatory, and antimicrobial properties). Nevertheless, identifying the compounds necessitates assessing various plant parts using diverse extraction methods to enhance the yield of particular phytochemical compounds. The following subsections delineate the primary chemical compounds in these plants.

#### Flavonoids

Various *Tamarix* species in Kazakhstan and China have presented flavonoids and their derivatives, such as *T. hispida* Willd., *T. hohenackeri* Bunge, *T. laxa* Willd., *T. ramosissima* Ledeb, and *T. elongata* Ledeb. The primary flavonoids indentified in this process include quercetin, kaempferol with its derivatives, and flavonoids associated with the genus *Tamarix* (tamarixetin and its derivatives) (Bikbulatova & Korul’kina, 2001; Xing et al., 2014; Umbetova et al., 2004a; Sultanova et al., 2002; Sultanova et al., 2004b; Utkin, 1966; Ren et al., 2019; Hong et al., 2018; Sultanova et al., 2001; Umbetova et al., 2005; Zhang & Tu, 1994). Interestingly, a distinctive compound isolated from *T. ramosissima* Ledeb. has also been reported as, 8-(1-(1-(3,4-dihydroxyphenyl)ethyl)-3′,4′-dimethoxy-3,5,7-trihydroxyflavone (ramosissimin) (Hong et al., 2018).

#### Phenolic acids

Considering the diverse biological activities of the genus *Tamarix,* phenolic acids serve as essential constituents in its phytochemical profiles. Previous studies asserted that phenolic acids were extracted from the aerial components of three species in Kazakhstan and China: (i) *T. hispida* Willd., (ii) *T. hohenackeri* Bunge, and (iii) *T. ramosissima* Ledeb. (Bikbulatova & Korul’kina, 2001; Xing et al., 2014; Ren et al., 2019; Sultanova et al., 2004a; Xiao et al., 2013). These studies identified the presence of gallic, ellagic, ferulic, caffeic, and protocatechuic acids within the various components with antioxidant and anti-inflammatory effects.

#### Triterpenoids

Triterpenes are abundant in *Tamarix* plants within Kazakhstan and China, such as *T. hispida* Willd., *T. hohenackeri* Bunge, *T. laxa* Willd., *T. ramosissima* Ledeb., and *T. elongata* Ledeb. (Xing et al., 2014; Sultanova et al., 2004b; Umbetova et al., 2004b; Sultanova et al., 2013). Specifically, a triterpenoid compound (isotamarixen) extracted from *T. hispida* Willd is a potential novel compound exhibiting antioxidant, anti-inflammatory, anti-tumor, and neuroprotective effects (Sultanova et al., 2004b).

#### Other phytochemicals

**Figure 2:**
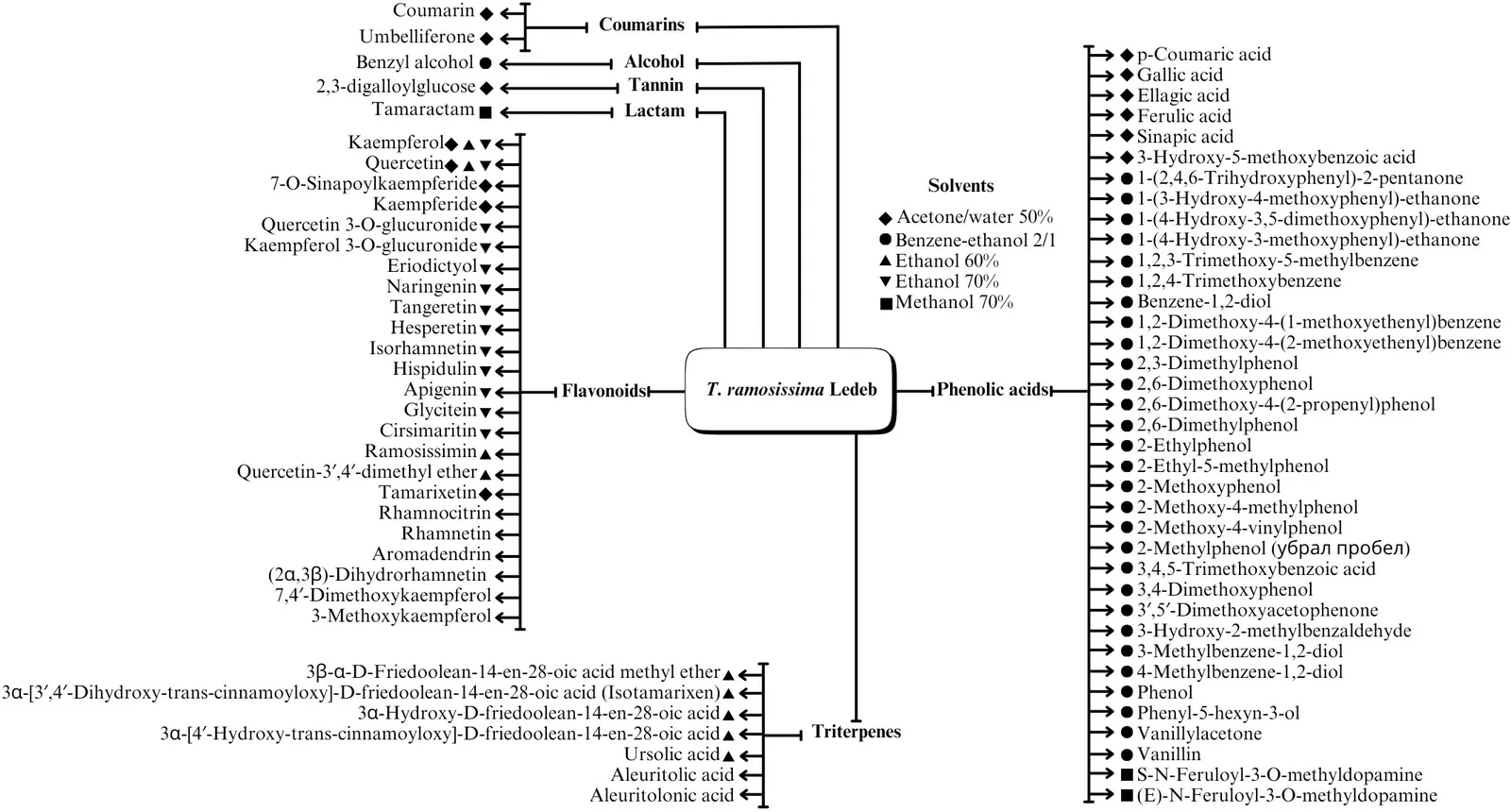
The chemical compounds of *T. ramosissima* Ledeb.

**Figure 3:**
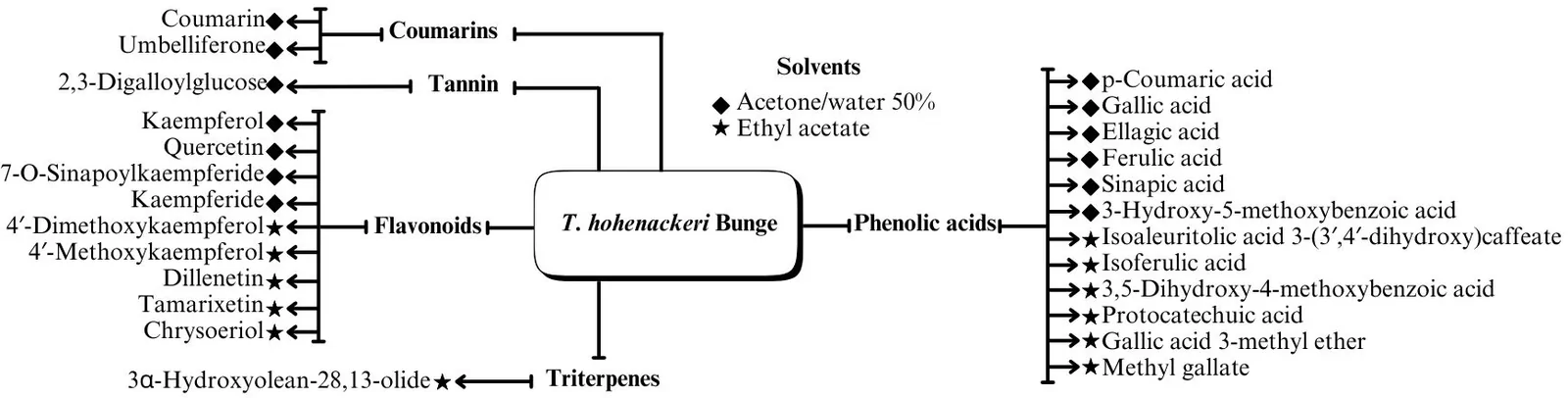
Chemical compounds *T. Hohenackeri* Bunge.

**Figure 4:**
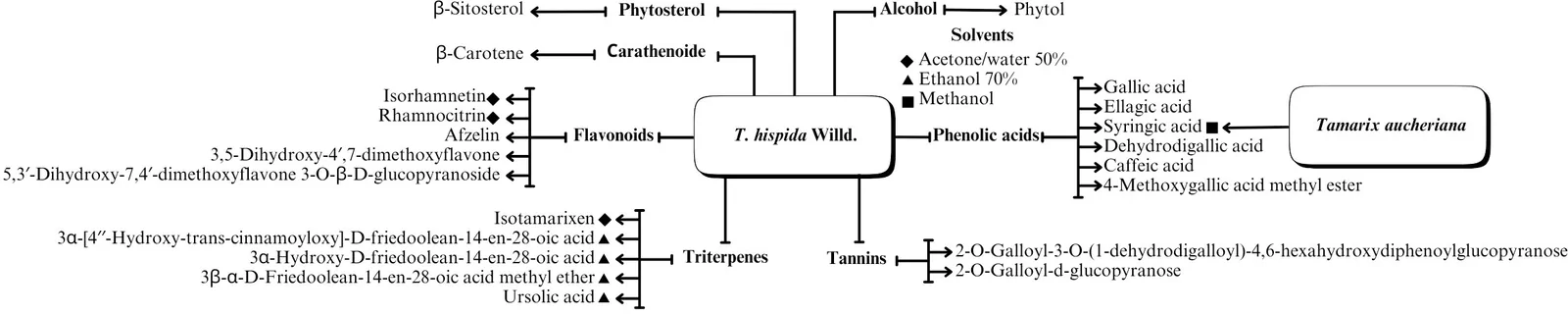
The chemical compounds of *T. Hispida* Willd.

**Figure 5:**
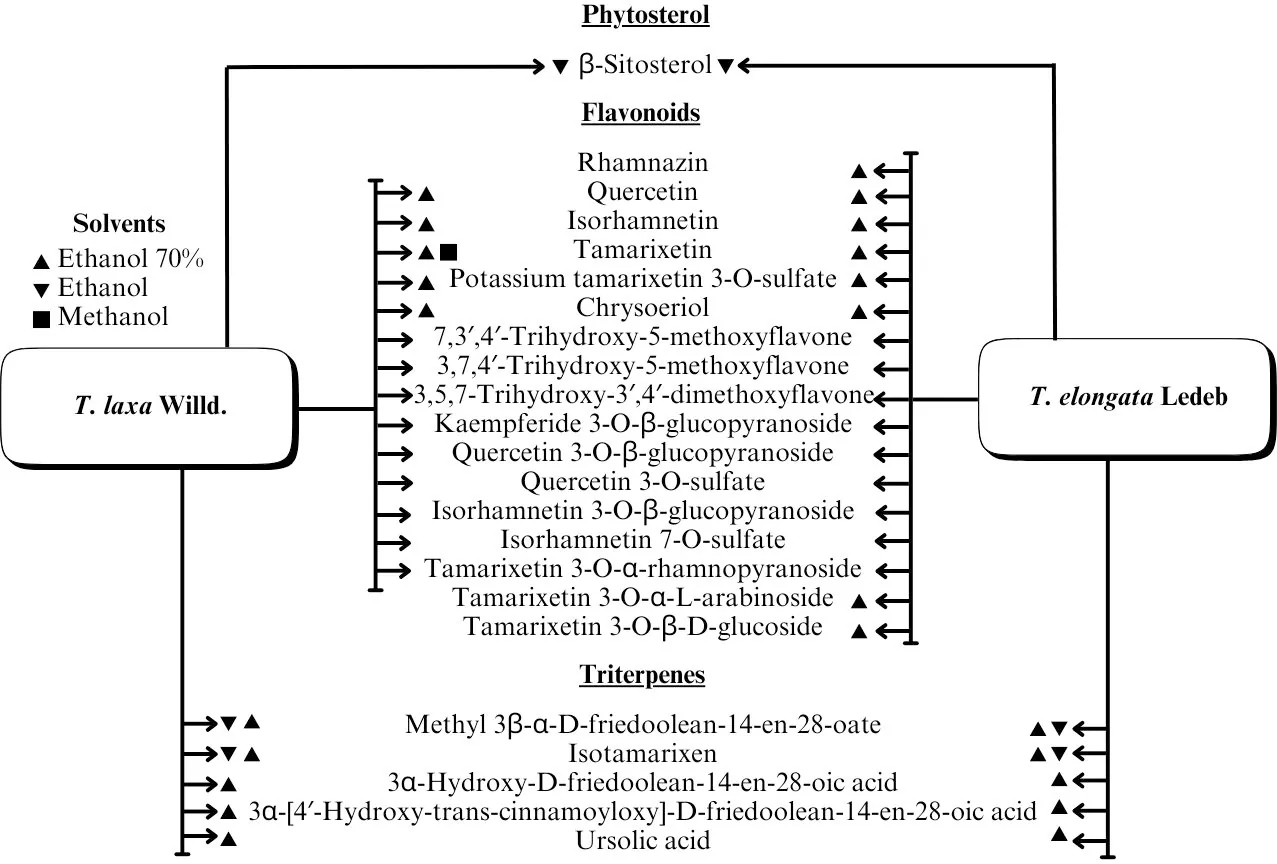
The chemical compounds of *T. Laxa* Willd. And *T. elongata* Ledeb.

A range of phytochemical constituents have been identified in *Tamarix* species, including alkaloids, coumarins, anthraquinone glycosides, lactams, tannins, tetraterpenes, and phytosterols (Xing et al., 2014; Orabi et al., 2013; Umbetova et al., 2004b). These compounds exhibit diverse pharmacological activities. Notably, the lactam derivative (*E*)-3-(4-hydroxy-3-methoxybenzylidene)-4-(4-hydroxy-3-methoxyphenyl) pyrrolidine-2-one (commonly referred to as tamaractam) was isolated from the branches and leaves of *T. ramosissima* Ledeb. (Yao et al., 2017). Therefore, phytochemicals demonstrate significant biodiversity, offering several potential applications in pharmaceuticals and medicine due to their complex interactions with biological systems. Figs. 2, 3, 4 and 5 provide an overview of the chemical compounds in the *Tamarix* genus.

#### Primary metabolites

Although most phytochemical investigations on *Tamarix* have focused on secondary metabolites, several studies have also explored the presence of primary metabolites, including amino acids, carbohydrates, vitamins, fatty acids, and minerals. These components not only play crucial physiological roles but also contribute to the adaptive potential of *Tamarix* species under extreme environmental conditions (Abilov & Sultanova, 2018).

#### Amino acids

The amino acid composition of *Tamarix* species—particularly *T. hohenackeri* Bunge and *T. ramosissima* Ledeb.—includes tryptophan, cysteine, threonine, glutamic acid, methionine, asparagine, glutamine, and arginine. Additional amino acids such as aspartic acid, serine, glycine, alanine, proline, tyrosine, valine, histidine, isoleucine, leucine, phenylalanine, and lysine have been identified in *T. ramosissima* (Abilov & Sultanova, 2018).

Kazakhstani studies further report the presence of 18 free amino acids in *T. elongata* Ledeb., *T. laxa* Willd., *T. ramosissima* Ledeb., and *T. hispida* Willd., with alanine, proline, leucine, and serine being predominant. The amino acid content was notably higher in samples collected from saline soils, suggesting a possible correlation with environmental salinity stress (Abilov & Sultanova, 2018).

#### Carbohydrates

The carbohydrate profiles of *T. elongata*, *T. laxa*, *T. ramosissima*, and *T. hispida* include monosaccharides such as glucose, galactose, rhamnose, and arabinose. Another study reported the presence of glucose, arabinose, xylose, galactose, and sucrose in *T. hohenackeri* and *T. ramosissima* (Abilov & Sultanova, 2018).

#### Vitamins

Vitamin analysis of *T. elongata*, *T. laxa*, *T. ramosissima*, and *T. hispida* revealed low levels of vitamin C, ranging from 0.003% to 0.046%. Vitamin E content in samples collected in the Almaty region ranged between 0.054% and 0.086% (Abilov & Sultanova, 2018).

#### Fatty acids

Fatty acid profiling of *T. elongata*, *T. laxa*, *T. ramosissima*, and *T. hispida* revealed a consistent qualitative composition across species, with 11 major saturated and unsaturated fatty acids identified. These include myristic, pentadecanoic, palmitic, palmitoleic, stearic, oleic, linoleic, eicosenoic, eicosadienoic, eicosatrienoic, and arachidonic acids. Quantitative variations among species were also noted (Abilov & Sultanova, 2018).

#### Mineral composition

Ash residue analysis of *Tamarix* species revealed the presence of essential macro- and microelements such as potassium, sodium, calcium, magnesium, copper, iron, zinc, cobalt, lead, nickel, and manganese. Sodium, potassium, and calcium were the most abundant macroelements (Abilov & Sultanova, 2018).

### Pharmacological attributes

*Tamarix* plants possess multiple biologically active attributes owing to their diverse chemical composition, including flavonoids, tannins, alkaloids, and essential oils. Various *Tamarix* species have also revealed significant antioxidant, antibacterial, anti-inflammatory, and antidiabetic activities (see Table 3).

**Table 3:** Pharmacological activity of the genus *Tamarix* of the flora of Kazakhstan.

**No**	**Pharmacological activity**	**Species of plant**	**Part of the plant**	**Extragent**	**The mechanism of action**	**Compound(s)**	**Ref**
1	**Antioxidant activity**	*Tamarix laxa* Willd., *Tamarix hispida* Willd., *Tamarix ramosissima* Ledeb., *Tamarix elongata* Ledeb.	Aerial parts	Ethanol, chloroform, ethyl acetate	Free radical scavenging	Ursolic acid, isotamarixen, pentacyclic triterpenoid, hispidulin, cirsimaritin and isorhamnetin	Ren et al. (2019); Sultanova et al. (2001); Sultanova et al. (2004a); Sultanova et al. (2013); Bukhari et al. (2021)
		*Tamarix ramosissima* Ledeb.		Ethanol (1:5)	Normalising the effects of oxidative stress in diabetes mellitus.	caffeic acid, lutheol, kaempferol chlorogenic acid, caffeic acid, rutoside	Rotaru et al. (2018)
2	**Antibacterial activity**	*Tamarix elongata* Ledeb, *Tamarix arceuthoides* Bunge, *Tamarix ramosissima* Ledeb, *Tamarix macrocarpa,* *Tamarix ramosissima*	Leaves	Ethanol, chloroform and n-hexane extracts	Suppression of bacterial colony growth, Inhibition of biofilms	gallic acid, isoferulic acid, kaempferol and quercetin, hispidulin, cirsimaritin and isoramnetin, polyphenolic compounds	Ren et al. (2019); Sultanova et al. (2001); Rotaru et al. (2018); Zazharskyi et al. (2020); Al-Shamma, Kadhum & Al-Hiti (2005); Akya et al. (2014); Rotaru & Varut (2020)
3	**Antimitogenic and chemosensitising activity**	*Tamarix passerinoides* Delile, *Tamarix aucheriana* *Tamarix elongata* Ledeb	Aerial parts	Methanol Petroleum ether	Induction of apoptosis, cell cycle alteration	Syringic acid, methyl ferulate	Abaza et al. (2013), Abaza et al. (2016)
4	**Antidiabetic activity**	*Tamarix hispida* Willd.	Aerial parts	Ethanol	Inhibition of enzymes associated with glucose metabolism	Hydrolysable tannins containing residues of gallic acid and its derivatives	Zhumagaliyeva et al. (2021)
5	**Anti-leishmanial activity**	*Tamarix ramosissima* Ledeb	Not specified	Chloroform, hydroalcoholic extract	Suppression of the viability of parasitic protozoa	Not specified	Qadir et al. (2014)
6	**Anti-inflammatory action**	*Tamarix hohenackeri* Bunge	Not specified	Not specified	Reduction in the production of inflammatory cytokines	Not specified	Chen et al. (2018)
7	**Inhibition of ACE and platelet aggregation**	*Tamarix hohenackeri* Bunge	Not specified	Ethyl acetate	Inhibition of angiotensin-converting enzyme and reduction of platelet aggregation	Flavonoids and phenolic compounds	Xing et al. (2014)
8	**Inhibitory effect on *α*-glucosidase activity**	*Tamarix hispida* Willd.	Aerial parts	Ethanol (50%)	Inhibition of *α*-glucosidase enzyme activity	Isotamarixen	Sultanova et al. (2013)
9	**Antiproliferative**	*Tamarix ramosissima* Ledeb *Tamarix elongata* Ledeb	Aerial parts	Ethanol Ligh petrol	Induces apoptosis in rheumatoid arthritis fibroblast-like synoviocytes, reducing the viability of synovial fibroblasts	Ramosissimin, methylferulate	Hong et al. (2018), Abaza et al. (2013)
10	**Antithrombolytic activity**	*Tamarix arceuthoides*		n-hexane	Platelet destruction		Bukhari et al. (2021)

#### Antioxidant properties

Antioxidant effectiveness is associated with considerable polyphenolic contents (such as flavonoids and tannins), which are known for their ability to neutralize free radicals and protect cells from oxidative stress. Significant examples include *T. laxa, T. elongata, T. hispida*, and *T. ramosissima,* possessing antioxidant attributes arising from terpenoid compounds (ursolic acid and isotamarixene) for capturing free radicals and preventing cell and DNA damage. The 2,2-diphenyl-1-picrylhydrazyl (DPPH) free radical capture method also presents that *T. ramosissima* extracts containing hispidulin and circymaritin exhibit potent antioxidant potential (Bahramsoltani et al., 2020). Furthermore, an inhibitory effect on *α*-glycosidase activity and significant antioxidant properties have been denoted in triterpenoid isotamarixene (3-*α*-[3, 4-dihydroxy-trans-cinnamoyloxy]-D-friedoolean-14-en-28-oic acids) within a 50% *T. hispida* ethanol extract (Sultanova et al., 2002; Ren et al., 2019; Li et al., 2024; Karker et al., 2024a).

The N-hexane extract of *T. arceuthoides* demonstrates protective effects on DNA from oxidative damage caused by H_2_O_2_, indicating notable antioxidant potential. This outcome signifies that the active compounds in the extract can inhibit or diminish oxidative damage to cellular structures. Multiple studies have also reported the antioxidant activities of other plant species for the genus *Tamarix*. This necessity has been attributed to the plant’s high levels of phenolic compounds and flavonoids. Consequently, these studies have stated that phenolic acids and flavonoids can effectively protect against oxidative stress, positioning them as promising candidates for medical applications to treat oxidative process-related diseases (neurodegenerative disorders and cardiovascular diseases). The data have successfully confirmed the significant potential of the *Tamarix* genus in developing natural antioxidant agents (Umbetova et al., 2004b; Sultanova et al., 2013; Yao et al., 2017; Bukhari et al., 2021; Zazharskyi et al., 2020; Gupta, Dubey & Kumar, 2016; Karker et al., 2024b; Zar Kalai et al., 2024; Samejo et al., 2023; Esma et al., 2023; Iqbal et al., 2022; Nisar et al., 2023).

#### Antibacterial activity

Different extracts of *Tamarix ramosissima* have shown varying antibacterial potential depending on the plant part and extraction solvent. A water–acetone leaf extract demonstrated *in vitro* activity against 15 microbial strains, with minimum inhibitory concentrations (MICs) ranging from 12.5 to 100 µg/mL and antifungal effects observed at 400 µg/mL. In contrast, the ethanolic bark extract was tested against seven bacterial and four fungal strains, showing MICs of 5–10 mg/mL and minimum bactericidal concentrations (MBCs) of 10–25 mg/mL. The bark extract exhibited stronger antibacterial activity against Gram-positive bacteria compared to Gram-negative ones (Bahramsoltani et al., 2020; Yusufoglu & Alqasoumi, 2011; Abo-Dola et al., 2015). The observed antibacterial effects may be attributed to the individual action or synergistic interactions of these identified compounds. Another example is the effective growth inhibition of various bacterial and fungal pathogens, such as *Corynebacterium diphtheriae and Aspergillus niger*, observed during *in vitro* assays using ethyl acetate and water-acetone extracts of *T. ramosissima*. These results highlight the potential these extracts as natural antimicrobial agents for combating infectious diseases (Rotaru & Varut, 2020).

Zazharskyi et al. (2020) argued that *T. elongata* extracts inhibited bacterial growth, including antibiotic-resistant pathogens. Based on the findings reported by Bukhari et al. (2021) the *n*-hexane extract of *T . arceuthoides* exhibited notable antibacterial activity, forming inhibition zones of 27.25 ± 0.37 mm against *Micrococcus spp.*, 14.75 ± 0.28 mm against *Staphylococcus aureus*, and 14.25 ± 0.24 mm against *Escherichia coli*. For comparison, the standard antibiotic erythromycin produced inhibition zones of 25.35 ± 0.26 mm, 30.25 ± 0.23 mm, and 34.75 ± 0.34 mm against the same bacterial strains, respectively. Although the extract showed a larger inhibition zone than erythromycin specifically against *Micrococcus spp.*, its overall efficacy was lower against the other tested strains.

In addition to antibacterial effects, *T. arceuthoides* extracts also demonstrated antibiofilm activity. The chloroform extract inhibited biofilm formation by *S. aureus* and *E. coli* by 47.36% and 45.53%, respectively, while the *n*-hexane extract produced slightly lower inhibition values of 45.92% for *S. aureus* and 38.58% for *E. coli*. In comparison, ciprofloxacin, used as a standard control, achieved significantly higher inhibition rates of 77.57% and 82.65%, respectively. These findings indicate that although *T. arceuthoides* extracts possess promising antibacterial and antibiofilm potential, their efficacy remains lower than that of standard antibiotics when assessed under controlled experimental conditions (Bukhari et al., 2021).

The antimicrobial activity of *T. macrocarpa* ethanol extract has been effectively evaluated, and it is active against *S. aureus*, *Bacillus subtilis*, and *Candida albicans*. This plant contains a high concentration of phenolic and polyphenolic compounds (gallic acid, isoferulic acid, campferol, and quercetin) based on the phytochemical analysis (Al-Shamma, Kadhum & Al-Hiti, 2005). Several studies have also computed a significant relationship between the antimicrobial properties of the genus *Tamarix* and the necessity of developing strategies to address pathogenic microorganisms regarding antibiotic-resistant forms. These studies have indicated that biologically active compounds in plants (flavonoids, phenolic acids, and tannins) demonstrate antimicrobial activity against bacteria and fungi. The antimicrobial activity of *Tamarix* is also influenced by its geographical origin and growing conditions, necessitating study to identify the most effective sources for pharmaceutical applications. Thus, studies concerning *Tamarix* species’ antimicrobial properties persist, presenting new opportunities for drug development to treat infectious diseases and applications within the pharmaceutical industry (Paul et al., 2022; El-Amier et al., 2021; Dawood, Al-Daody & Al-Takay, 2021; Romeilah et al., 2021; AbouZid & Sleem, 2011; Al-Omar et al., 2020; Boulaaba et al., 2015; Lefahal et al., 2010).

#### Antimitogenic and chemosensitizing activity

Abaza et al. (2013) indicated that syringic acid and methyl ferulate isolated from methanolic *T. passerinoides* Delile and *T. aucheriana* extracts could be adjuvant agents in colorectal cancer management (Lefahal et al., 2010; Al-Judaibi, 2020). The studies explained that the primary actions of these compounds included inhibiting tumor cell growth, inducing apoptosis, and enhancing cell sensitivity to chemotherapeutic agents. A positive correlation between syringic acid and chemotherapy efficiency was also reported. Peydayesh et al. (2020) investigated the inhibitory effect of silver nanoparticles from *T. hispida* extracts on the cyclin D1 protein expression in human cancer cell lines. The study described the methodology based on biosynthesized silver nanoparticles for evaluating the anti-cancer effect concerning molecular mechanisms, cell cycle regulation, and cancer cell proliferation. This method offered an alternative approach to nanoparticle production that eliminated chemicals commonly employed in nanomaterial synthesis. Therefore, the biological synthesis method could reduce toxicity and impart additional biologically active properties to nanoparticles due to compounds present in extracts (Aljubouri & Alobaidi, 2023).

Numerous studies on the *Tamarix* genus have garnered scientific interest regarding its potential anti-cancer properties. These studies demonstrate that *Tamarix* extracts are active against various cancer cell types, including breast, lung, and colon cancers. The anti-tumor effects observed in laboratory conditions also indicate a growth reduction of cultured malignant cell tumors, suggesting that these plants are promising candidates for pre-clinical studies (Abaza et al., 2013; Abaza et al., 2016; Lefahal et al., 2010; Al-Judaibi, 2020; Peydayesh et al., 2020; Nicolini et al., 2014; Abdel-Tawab et al., 2019; Boulaaba et al., 2013).

#### Antidiabetic activity

Given the increasing prevalence of diabetes (particularly type 2 diabetes) involving insulin resistance and metabolic disorders, studies regarding the antidiabetic properties of the genus *Tamarix* have attracted interest from the scientific community. Plants within this genus usually contain various biologically active compounds that can positively influence glucose and lipid metabolisms. Specifically, *T. hispida* possesses tannins inhibiting the enzyme *α*-glucosidase, preventing a rapid increase in blood sugar levels (AbouZid & Sleem, 2011). Specific antidiabetic property-related studies of extracts from several *Tamarix* genera have also demonstrated their capacity to reduce blood glucose levels in experimental animals. This outcome is attributed to improved insulin sensitivity and increased glucose absorption capacity of tissues (Alnuqaydan & Rah, 2021; Alnuqaydan et al., 2022; Fayed et al., 2023; Al Sobeai, 2018; Sehrawat & Sultana, 2006; Ullah et al., 2017; Hebi & Eddouks, 2017; Mahfoudhi et al., 2016).

#### Anti-inflammatory action

Chen et al. (2018) assessed the anti-inflammatory properties of *T. hohenackeri* extracts. The study identified potential neuroinflammation inhibitors from four *T. hohenackeri* extracts while examining their possible mechanisms of action. Consequently, reduced inflammatory response was observed due to the flavonoids and phenolic acids reducing the cytokine production in lipopolysaccharide-activated microglia. Previous studies also examined the anti-inflammatory properties of other *Tamarix* species (Zhumagaliyeva et al., 2021; Chen et al., 2018; Yusufoglu & Alqasoumi, 2011; Fayez et al., 2024; Hamed et al., 2024). These studies indicated that the main mechanisms of their action involved inhibiting inflammatory mediators and lowering inflammatory cells’ activity. The *Tamarix* plant extracts were also reported as adjunctive treatments (inflammatory conditions) and aided formulation of phytotherapeutic agents (anti-inflammatory and antioxidant properties) owing to their capacity to suppress the activity of pro-inflammatory cytokines and enzymes while reducing oxidative stress,

#### Inhibition of ACE and platelet aggregation

Xing et al. (2014) investigated bioactive compounds isolated from *T. hohenackeri* Bunge a species traditionally used in folk medicine and known for its for its radioprotective properties. The study suggested that flavonoids and phenolic compounds present in the extract exhibited inhibitory effects on angiotensin-converting enzyme (ACE) and platelet aggregation, indicating potential cardiovascular benefits. In another study, Bukhari et al. (2021) evaluated the thrombolytic activity of *T. arceuthoides in vitro*. The experiment involved incubating human blood clots with plant extracts at 37 ^∘^C and measuring the percentage of clot lysis. Streptokinase served as the positive control, showing 70.83 ± 0.84% clot lysis. Among the tested extracts, the n-hexane extract of *T. arceuthoides* demonstrated thrombolytic activity of 19.72%, whereas the chloroform extract exhibited 18.57%. Although the activity was lower compared to the standard, these findings suggest the presence of bioactive compounds with potential clot-dissolving properties that warrant further investigation.

#### Antileishmanial activity

Akya et al. (2020) investigated the antileishmanial effects of hydroalcoholic and chloroformic extracts of *Tamarix ramosissima* against *Leishmania major* and *Leishmania tropica* promastigote forms. The hydroalcoholic extract reduced parasite viability at all tested concentrations, with an IC_50_ value of 0.1 mg/mL for both species. The chloroformic extract showed moderate inhibitory effects, with a measured IC_50_ of 50 mg/mL. These findings indicate that *T. ramosissima* exhibits high antileishmanial activity and holds promise as a natural source for developing novel agents against leishmaniasis (Akya et al., 2020).

#### Antiproliferative and apoptosis-inducing effects

The tamaractam and ramosissimin compounds isolated from *T. ramosissima* induce apoptosis while inhibiting the growth of synoviocytes associated with rheumatoid arthritis (Umbetova et al., 2004a; Hong et al., 2018; Yao et al., 2017).

## Conclusions

The study of 13 *Tamarix* species found in Kazakhstan has shown that species such as *T. ramosissima*, *T. hispida*, and *T. laxa* have been more extensively studied compared to less explored species like *T. eversmannii*, *T. meyeri*, and *T. arceuthoides*. Further research is needed to conduct a detailed analysis of their morphological, molecular phylogenetic, and chemotaxonomic characteristics. Among the identified biological activities, the most promising ones include antioxidant, antibacterial, anti-inflammatory, antidiabetic, and anticancer properties. The antioxidant activity is attributed to the high content of polyphenols and flavonoids, making *Tamarix* a potential source of natural antioxidants for medical and food applications. The antibacterial activity, particularly pronounced in the bark and leaf extracts of *T. ramosissima*, suggests potential for developing novel natural antimicrobial agents. The anti-inflammatory and antidiabetic properties indicate the potential of *Tamarix* for developing phytopharmaceuticals, while specific compounds such as tamarixetin and tamaractam may be utilized in cancer therapy.

Potential areas of application include pharmacology, agriculture, and the food industry. In medicine, *Tamarix* can be used to develop treatments for inflammatory and metabolic diseases. In agriculture, its high tolerance to drought and salinity makes it a valuable resource for soil stabilization and land restoration. In the food industry, *Tamarix* extracts may serve as natural antioxidants and functional additives.

To resolve controversial taxonomic issues, it is recommended to use molecular genetic methods (ISSR, RAPD, ITS, SNP markers) to clarify phylogenetic relationships and distinguish closely related species, apply DNA barcoding to identify hybrid forms, conduct comprehensive morphological and anatomical studies, including palynomorphological analysis, and perform chemotaxonomic analysis based on secondary metabolite profiles. The development of a unified database integrating molecular, morphological, and chemical characteristics of *Tamarix* species in Kazakhstan will enable the standardization of their identification and classification criteria. Further research will not only help eliminate existing gaps in the study of these plants but also expand their practical applications in various scientific and industrial fields.

It is also important to highlight that *T. androssowii*, one of the species distributed in Kazakhstan, is listed in the Red Book of Kazakhstan (2014) as a rare and endangered species. This fact underscores the dual importance of the genus *Tamarix*—not only for the development of phytopharmaceuticals and other practical applications but also for biodiversity conservation and sustainable use of plant resources in Kazakhstan’s arid and semi-arid ecosystems.

## Additional Information and Declarations

### Data Availability

The following information was supplied regarding data availability: This is a literature review.
